# Study on the Multi-level Resistance-Switching Memory and Memory-State-Dependent Photovoltage in Pt/Nd:SrTiO_3_ Junctions

**DOI:** 10.1186/s11671-018-2433-5

**Published:** 2018-01-12

**Authors:** Shengkai Wang, Xianwen Sun, Guanghui Li, Caihong Jia, Guoqiang Li, Weifeng Zhang

**Affiliations:** Henan Key Laboratory of Photovoltaic Materials and School of Physics and Electronics, Henan University, Kaifeng, 475004 People’s Republic of China

**Keywords:** Resistive switching, Interface state, Multi-level memory, Photovoltage

## Abstract

Pt/Nd:SrTiO_3_ (STO)/In devices were fabricated by depositing Schottky-contact Pt and Ohmic-contact In electrodes on a single crystal STO with Nd doping. The Pt/Nd:STO/In devices show multi-level resistance-switching (RS) memory and memory-state-dependent photovoltage (PV) effects, which can be controlled by the applied pulse width or magnitude. Both the RS and PV are related to the bias-induced modulation of the interface barrier, both in height and width, at the Pt/Nd:STO interface. The results establish a strong connection between the RS/PV effects and the modulation of the Nd:STO interface triggered by applied electric field and provide a new route by using an open-circuit voltage for non-destructively sensing multiple non-volatile memory states.

**Electronic supplementary material:**

The online version of this article (10.1186/s11671-018-2433-5) contains supplementary material, which is available to authorized users.

## Background

SrTiO_3_ (STO) is a large bandgap (3.2 eV) insulator. It is considered to be a model perovskite material due to its simple cubic structure in a wide temperature range [1]. STO has abundant photoelectric performance which can be directly manipulated via doping with a donor- or acceptor-type transition metal. The range of applications of STO system is very wide [2, 3]. Recently, STO system has received a great deal of scientific attention due to the resistive switching (RS) phenomena, which can be considered as a good candidate for building the resistive random access memory (RRAM) [4, 5].

The RS device based on STO is usually a metal/STO/metal structure. The RS properties of STO system, i.e., from acceptor- to donor-doped STO, have been widely investigated. Different physical mechanisms have been proposed to explain the switching behavior. For acceptor-doped (e.g., Fe and Cr) STO, the works emphasize the property change in the crystal bulk, in which the RS was attributed to electric-field-driven migration of oxygen vacancy, either the fast transport of oxygen vacancies along dislocations or the formation of oxygen-vacancy array under high electrical stress [6–11]. On the other hand, for RS device based on donor-doped (e.g., Nb) STO, the Schottky-type contact between metal and n-type Nb:STO is necessary and emphasized by many works. However, some reports have connected RS with changes of the electron depletion layer in metal/Nb:STO junctions, which is caused by the oxygen stoichiometry within a thin interfacial layer [12–14] or by a deviation from the nominal cation stoichiometry in the near-surface region [15, 16] and some reports suggest that the interfacial barrier is kept unchanged during RS process, but conductive filaments play a vital role for the resistance change [17–19].

In view of the above reported points, it is obvious that there has been no consensus about the switching mechanism of donor-type STO. Two views of interface and bulk resistance change coexist so far. As for the specific reasons for RS, there are still many reported physical mechanisms. The unclear physical mechanism stands in the way of progress of RRAM based on STO material. For clarifying the RS mechanism and developing RRAM device based on donor-type STO system, it is advantage to study different metal-doped STO material.

The electronic transport properties of STO can be modulated via doping with transition metal [20]. Compare with thin films, single crystal have homogeneous properties over the entire area and well-established defect physics and chemistry. Up to now, we only found donor-doped STO single crystals with Nb element reported for RS devices. For Nd-doped STO single crystals (Nd:STO), the ionic radius of Sr^2+^, Ti^4+^, and Nd^3+^ (Nd^2+^) is 0.118, 0.0605, and 0.0983 (0.129) nm, respectively, suggesting that Nd^3+^ could easily substitute Sr^2+^ rather than Ti^4+^ due to similar radius between Nd^3+^ and Sr^2+^ [21]. This substitution site is different from n-type Nb:STO. So, Nd:STO single crystal is a donor-doped material and at n-type conductivity which will be certified by Hall effect later. Nd:STO single crystal is a new n-type STO for RS, and we did not find the reported works so far.

It is generally known that the photovoltaic (PV) effect is related to the internal electric field [22–26]. So, the PV effect is expected to be dependent on the memory states if the RS is mainly determined by the depletion layer near the metal and n-type STO interface. Conversely, the PV is irrelevant to the memory states if the RS is induced by the conductive filaments. In this work, we fabricated Schottky-contact Pt and Ohmic-contact In electrodes on n-type Nd:STO single crystal. The RS memory and PV effect were studied together to clarify the switching mechanism of Pt/Nd:STO/In device. Interestingly, the results clearly show that the Pt/Nd:STO/In device has multi-level memory and memory-state controlled PV effect, which can be modulated by the switching bias. The results suggest that the shared mechanism for RS and PV relates to the modulation of the Pt/Nd:STO interface barrier, which are induced by the injection and trapping or detrapping of carriers.

## Methods

Single crystals Nd:STO (100) in size of 5 mm **×** 5 mm **×** 0.5 mm with 0.05 wt% Nd doping were selected as substrate. The In electrodes (orange electrodes) were directly pressed on the rough surface of Nd:STO to form the Ohmic contacts. The Pt electrodes with a diameter of 0.1 mm were sputtered onto the Nd:STO single crystal through a shadow mask (blue electrodes). The distance between two close Pt electrodes was 0.5 mm. The inset in Fig. 2a shows the configuration of Pt/Nd:STO/In and In/Nd:STO/In devices. The current-voltage (I–V) and RS characteristics were measured on a Keithley 2400 SourceMeter. A positive electrical field is defined as the current flowing from the In to Pt electrode.

Hall effect was carried out using Ecopia HMS-3000 Hall measurement system in order to investigate the carrier concentration induced by the Nd doping. The crystalline structure of the STO was examined by x-ray diffraction (XRD, Bruker, D8-Advance) using Cu Kα radiation. Raman scattering measurements were carried out on a confocal micro-Raman spectrometer (Renishaw R-1000) with visible laser light of wavelength 632.8 nm as an excitation source.

## Results and Discussion

**Fig. 1:**
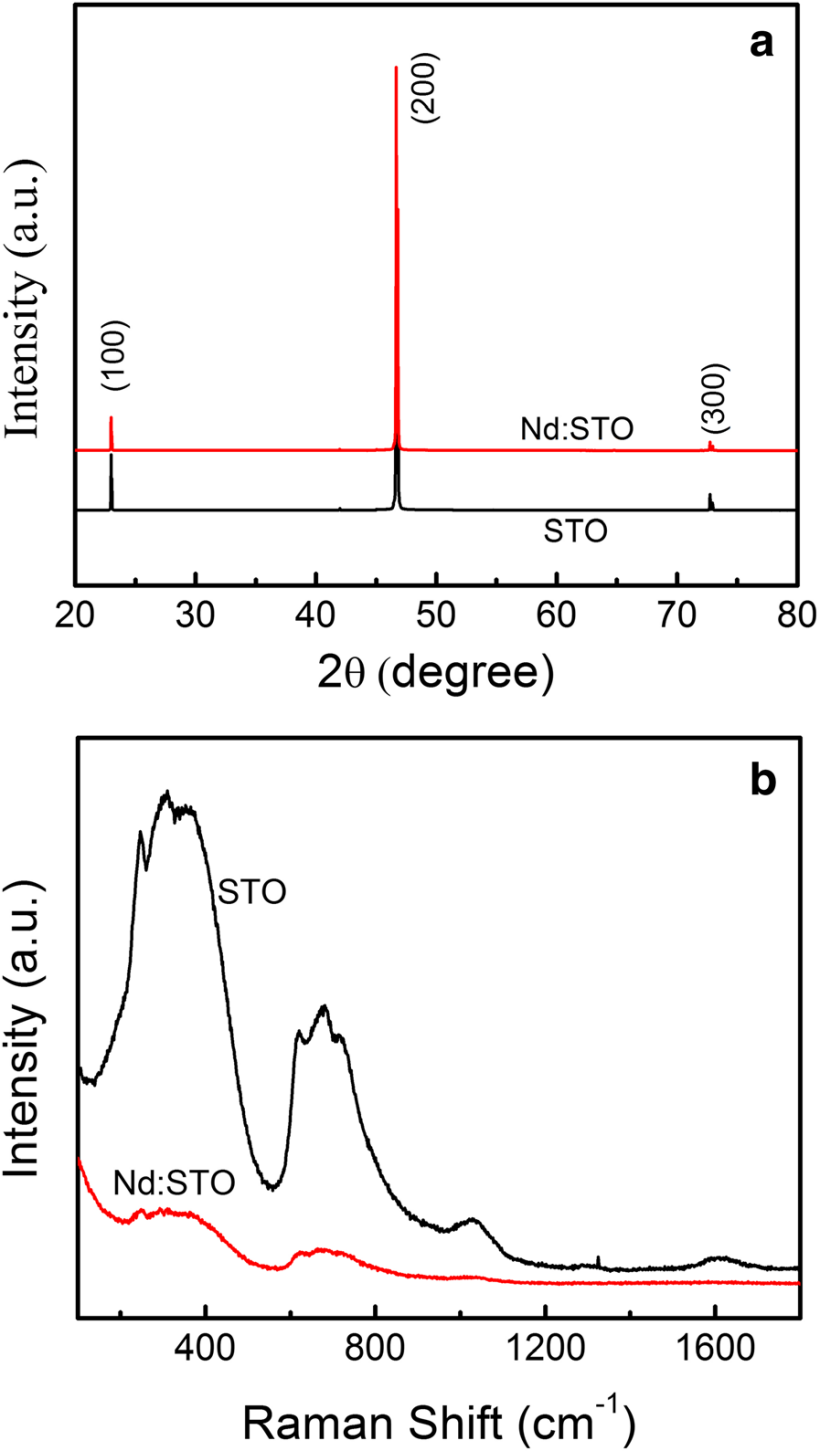
**a** The XRD patterns and **b** Raman spectra of undoped STO and Nd-doped STO single crystal

Figure 1a shows the XRD patterns of undoped STO and Nd:STO single crystals. All peaks are corresponding to the perovskite phase and can be indexed to the cubic space group Pm3m with lattice constant a ≈ 3.905 Å. The peaks do not show any observable change after Nd implantation, indicating that the Nd doping has little effect on the bulk structure. The Raman spectra of undoped STO and Nd:STO single crystals are given in Fig. 1b. The Raman spectra of undoped STO shows two distinct broad bands originating from second-order scattering, which are centered at 200–400 cm^−1^ and 600–800 cm^−1^ and belongs to ideal cubic perovskite structure. The position of these two bands is in agreement with the published literature [27, 28]. The broaden line with decreased second-order broad band in Nd:STO is also observed, indicating a weaker centrosymmetry as a result of local disorder induced by Nd doping. Comparing with the XRD patterns, the Raman results indicate that there exist some structural defects on the surface of Nd:STO single crystal, which should be induced by the Nd doping.

It is generally known that the undoped STO single crystal is an insulating material. To investigate the Nd-doping impact on the electrical properties of STO single crystal, Hall effect was measured. The Hall results shows that the Nd:STO single crystal is at n-type conductivity, and the carrier concentration is about 2 **×** 10^19^ cm^−1^. This n-type conductivity can be attributed to the substitution of Nd^3+^ into Sr^2+^ sites.

**Fig. 2:**
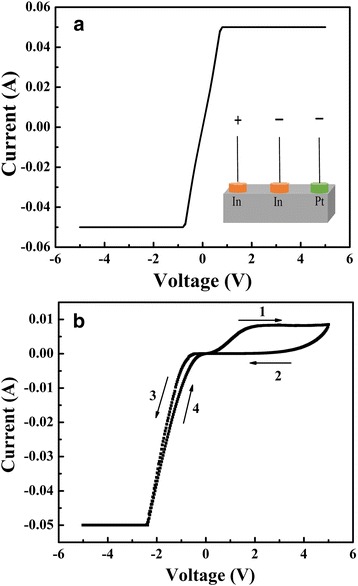
The *I*–*V* characteristics of **a** the In/Nd:STO/In and **b** Pt/Nd:STO/In devices in the voltage range from 0 V → 5 V → 0 V → − 5 V → 0 V with 50-mA compliance current. The inset shows the device schematic illustration

The inset of Fig. 2a shows the schematic illustration of In/Nd:STO/In and Pt/Nd:STO/In devices. The *I*–*V* characteristics of both In/Nd:STO/In and Pt/Nd:STO/In devices are plotted in Fig. 2a, b, respectively. The sweeping voltage was applied as 0 V → 5 V → 0 V → − 5 V → 0 V with a 50-mA compliance current. The In/Nd:STO/In device has linear *I*–*V* curves (shown in Fig. 2a) and exhibits a good Ohmic contact between the pressed In electrodes and Nd:STO single crystal, but no RS effect appears, while the Pt/Nd:STO/In device shows reversible RS properties, as shown in Fig. 2b. When the applied voltage increases, the resistance transition occur, the direction of transition depends on the polarity of applied voltage. When the applied voltage decreases, the high- and low-resistance state (HRS and LRS) will be maintained, indicating that the resistance state is stable and nonvolatile after the formation. The large *I*–*V* hysteresis shows the Pt/Nd:STO/In device has the memory properties; the prototypical diode behavior indicates that a Schottky barrier is formed at the Pt and n-type Nd:STO interface and dominates the resistance of Pt/Nd:STO/In device. Therefore, it is easy to conclude that the RS effect of Pt/Nd:STO/In device comes from Schottky interface between Pt and Nd:STO single crystals. This result, RS is dependent on Schottky interface, is in agreement with our reported works on n-type Nb:STO single crystal [29].

**Fig. 3:**
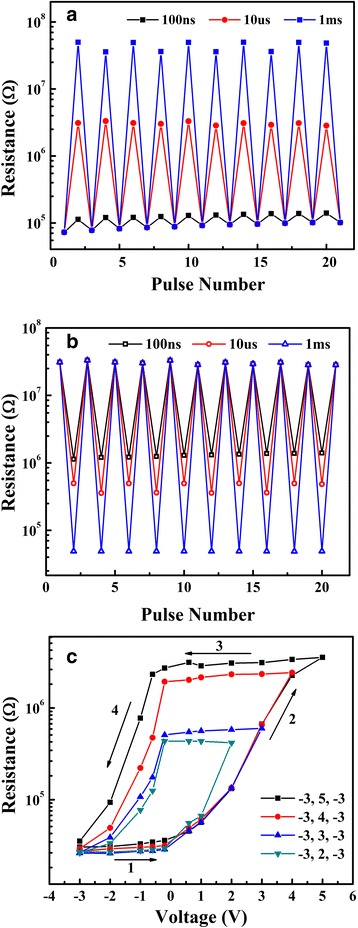
Consecutive RS cycles **a** from LRS to HRS and **b** from HRS to LRS. The device was firstly set to LRS (HRS) by a − 5 V (+ 5 V) pulse with 100 ms width and then applied by a + 5 V (− 5 V) pulse with varied pulse widths of 100 ns, 10 μs, and 10 ms, respectively. The corresponding resistance transition from LRS (HRS) to intermediate resistance states or HRS (LRS). **c** *R*–*V* hysteresis loops controlled by pulse voltage. The Pt/Nd:STO/In device was firstly set to LRS by a pulse of − 3 V, followed by sweeping the pulse to + 2 V (or + 3, + 4, and + 5 V) and back to − 3 V with 100-ms pulse width. All the resistance was read at 0.1 V

To evaluate the potential for application of the Pt/Nd:STO/In device in multi memory, the effect of the pulse width and amplitude on the resistance states was investigated and shown in Fig. 3a–c. The device was firstly set to LRS by a − 5-V pulse with 100 ms width and then applied by a + 5-V pulse with varied pulse widths of 100 ns, 10 μs, and 10 ms, respectively. The resistance was read at 0.1 V. The corresponding resistance transition from LRS to intermediate resistance states or HRS was achieved, as shown in Fig. 3a. Figure 3b shows the consecutive RS cycles from HRS to LRS induced by the opposite polarity pulses. The results confirm that the multi-level resistance can be obtained by pulse voltage with different widths. The retention property of each resistance state was further investigated, and no significant change in resistance magnitudes was observed (shown in Additional file 1: Figure S1). Figure 3c presents typical non-volatile resistive memory loops controlled by pulses voltage. The Pt/Nd:STO/In device was firstly set to LRS by a pulse of − 3 V, followed by sweeping the pulse voltage to + 2 V (or + 3, + 4, and + 5 V) and back to − 3 V with 100-ms pulse width. The resistance was read at 0.1 V. A series of intermediate resistance states can be achieved by adjusting the pulse magnitude. From the Fig. 3a–c, we have the result that multi-level resistance state of Pt/Nd:STO/In device can be achieved by adjusting the pulse width or magnitude, indicating that the device behaves as a memristor [23, 30].

**Fig. 4:**
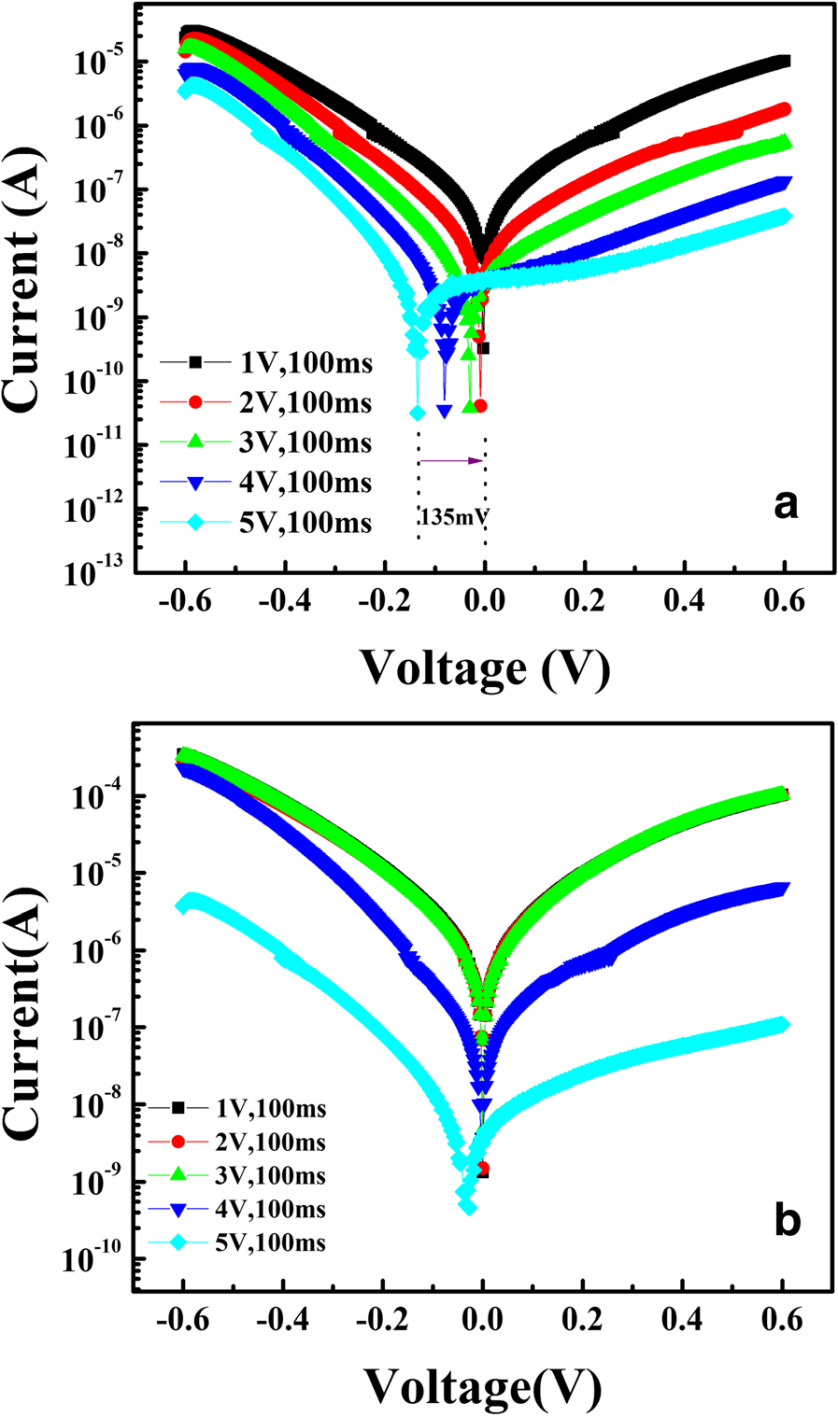
The *I*–*V* curves in the low-bias regime (− 0.6 to + 0.6 V) after switching with a series of voltage pulses from + 1 to + 5 V with 100 ms (switching from LRS to intermediate resistance states and to HRS) under **a** the light illumination and **b** dark, respectively

It is generally known that photoexcitation of charge carriers occurs when the illuminated wavelength matches the optical bandgap of the active material. The generated electrons and holes will be separated by the internal electric field, which leads to the PV effect [23–25]. In the case of Pt/Nd:STO/In device, if the multi-level memory states are mainly determined by the depletion layer near the Pt/Nd:STO interface, the PV effect is expected to be dependent on the memory states of the device. On the contrary, the PV is irrelevant to the memory states if the depletion layer is kept unchanged during the RS process. More interestingly, we found a memory-state-dependent PV effect for the Pt/Nd:STO/In device. Figure 4a, b shows the *I*–*V* curves in the low-bias regime (− 0.6 to + 0.6 V) after switching with a series of pulses from + 1 to + 5 V with 100 ms (switching from LRS to intermediate resistance states and to HRS) under the light illumination and dark, respectively. Under light illumination, the *I*–*V* curves of HRS exhibit notable shifts along the voltage axis, and the open-circuit voltage (Voc) (voltage at zero current) is as high as ~ 135 mV. Corresponding to a series of intermediate resistance states, the Voc gradually decreases with decreasing device resistance and is negligible small for LRS. Whereas little shift has been observed for *I*–*V* curves measured in the dark. The similar result was obtained by Hu et al. [23]. Furthermore, a test method for Voc was reported by Shang et al. [24–26]. According to this method, the Voc was further measured at LRS and HRS. As expected, a voltage rise is produced by light illumination, and the Voc is dependence on the junction resistance (see Additional file 1: Figure S2). The above results testify that the magnitude of Voc depends on the memory states of Pt/Nd:STO/In device.

**Fig. 5:**
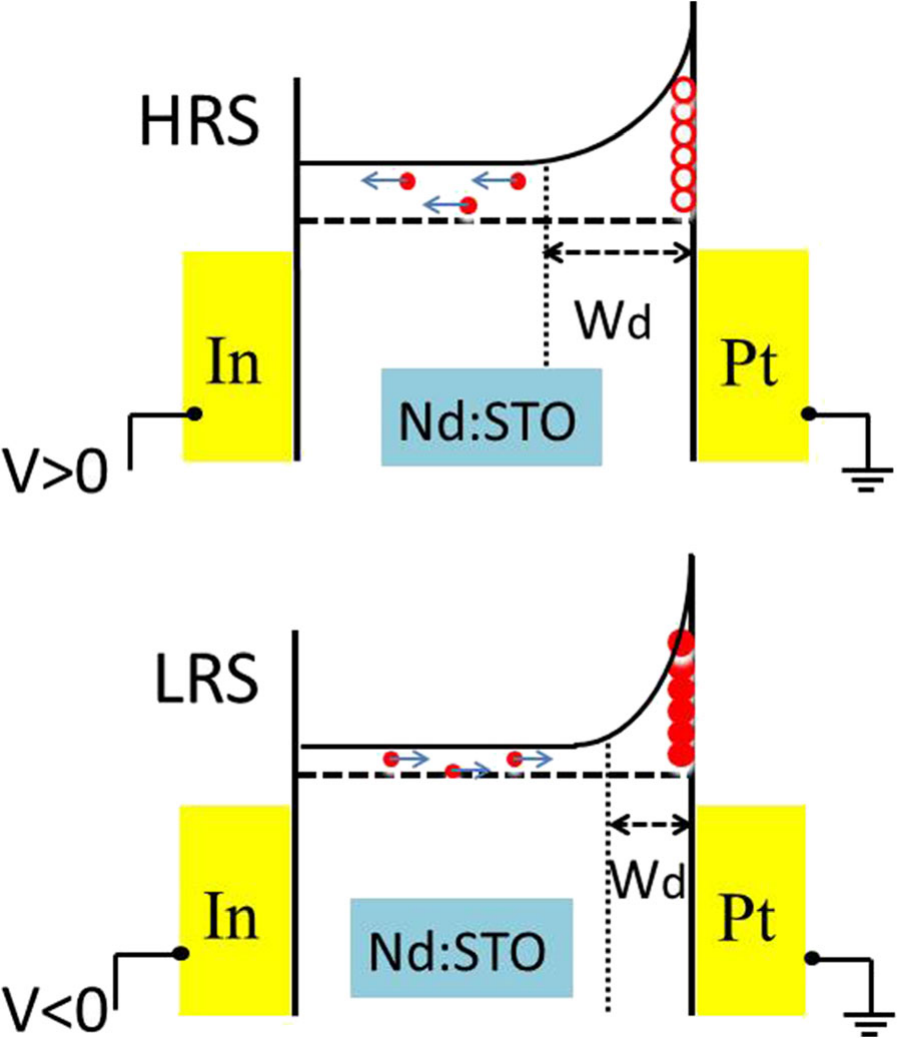
Schematic diagram of energy band structure and interface state Pt/NSTO/In system at HRS and LRS. The red hollow and solid spheres at interface represent the unoccupied and occupied interface state, respectively

The multi-level memory and memory-state-dependent PV effect of Pt/Nd:STO/In device unambiguously suggest that the memory states are mainly determined by the depletion layer near the Pt/Nd:STO interface. In other words, the Schottky barrier width and height near the Pt/Nd:STO interface will be modulated by sweeping voltage. According to the Raman results in Fig. 1b, there exist some defects (e.g., oxygen vacancies) at the Nd:STO surface. When a negative voltage or pulse was applied to the device, the injected electrons from the In electrode were trapped by the defects at the Pt/Nd:STO interface. The trapped electrons lead to narrower and lower Schottky barrier, resulting in LRS. In contrast, during the coming positive bias sweeping, the trapped electrons are released because of the existence of the depletion region, resulting in HRS. Furthermore, the spatial distribution of defects should be uneven. Fermi pinning may be formed at the high density defect, so the HRS and LRS can be kept when the applied bias is removed. The depletion layer can be adjusted by the pulse width or magnitude, so multi-level memory states were observed. Figure 5 shows the schematic diagram for the process of the electron trapping or detrapping on the Pt/Nd:STO interface.

The memory-state-dependent PV effect is induced by different width and height of the Pt/Nd:STO interface barrier at different memory state. The HRS with wider depletion region results in more electron-hole pairs generated in depletion region under light illumination. The photogenerated electrons is driven into the Nd:STO bulk by the strong upward band bending in the HRS, and the holes tunnel through the barrier, resulting in a higher Voc. Conversely, the LRS with lower and narrower depletion region results in a lower Voc. In general, the Voc is dependent on interface barrier width and height which is corresponding to the multi-level memory states of Pt/Nd:STO/In device.

Note that both the memory states and the PV effect exhibit similar dependence on the switching bias, indicating the shared mechanism related to the electron depletion/accumulation on the Nd:STO interface, revealing the importance of the interface barrier and interface charge redistribution (Fig. 5). The PV effect is induced by the photogenerated electrons and holes separated by the internal electric field. So, the memory-state-dependent PV effect observed in Pt/Nd:STO/In device testify the RS is induced by the bias-induced modulation of Schottky barrier on Pt/Nd:STO interface and not by the formation of conductive filaments. The Voc is dependent on the memory states, so such a resistance-state-dependent PV effect provides a new route by using Voc for sensing the memory states of RS device in addition to the conventional resistance reading [23]. This new reading route is non-destructive and reliable because light illumination will not change the memory state of the devices.

## Conclusions

In summary, we have investigated the RS and PV characteristics of single crystalline Nd:STO-based memristive devices. The RS effect is related to the Schottky junction near the interface of Pt and n-type Nd:STO single crystal. The memory states can be modulated by the pulse width or magnitude. The memory-state-dependent PV effect of the Pt/Nd:STO/In device is obtained by the switching voltage. These complimentary effects are attributed to the bias-induced modulation of the interface barrier, both in height and width, at the Pt/Nd:STO interface, which is caused by carrier injection and trapping/detrapping process on the Pt/Nd:STO interface. The results establish a strong connection between the RS/PV effects and the modulation of the Nd:STO interface triggered by applied electric field and provide a new route by using Voc for non-destructively sensing multiple non-volatile memory states.

## Additional file

Additional file 1: Supporting information. (DOCX 154 kb)

## Abbreviations

HRS: High-resistance state
*I*–*V*: Current-voltage
LRS: Low-resistance state
PV: Photovoltage
RRAM: Resistance random access memories
RS: Resistance switching
XRD: X-ray diffraction
