# Supplementary material for: Study on the Multi-level Resistance-Switching Memory and Memory-State-Dependent Photovoltage in Pt/Nd:SrTiO3 Junctions
Source: Nanoscale Res Lett. 2018 Jan 12;13:18. doi: 10.1186/s11671-018-2433-5 (PMC5766446; doi:10.1186/s11671-018-2433-5)
Supplement: Additional file 1: — Supporting information. (DOCX 154 kb) [file 11671_2018_2433_MOESM1_ESM.docx]

**Surporting information**


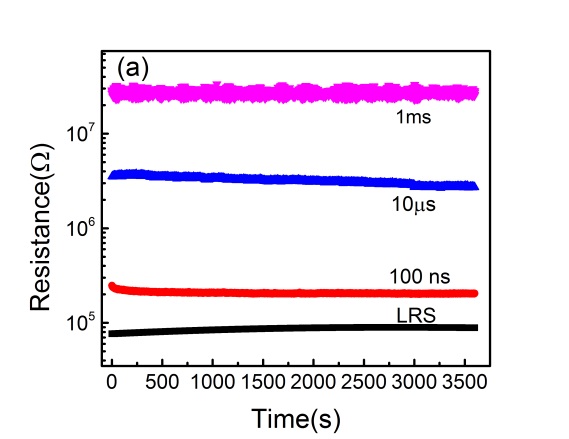

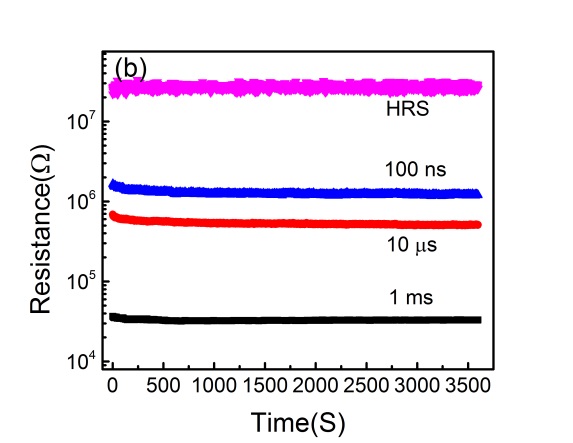


Figure S1 The corresponding retention property of each resistance states in Fig.3a and 3b. No significant change in resistance magnitudes was observed, indicating that the resistance state is nonvolatile.


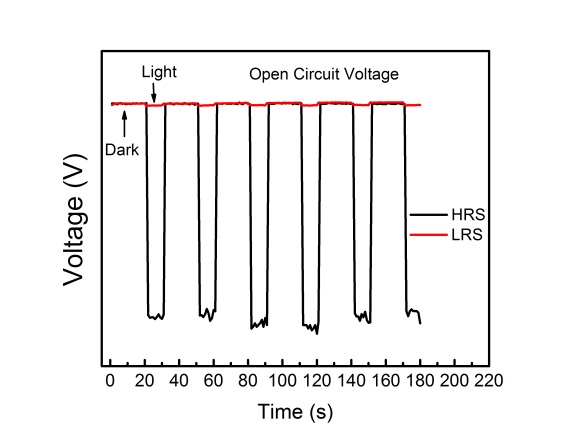


Figure S2 The Voc was measured at LRS and HRS under the light illumination and dark, according to the test method reported by Shang et al. [1-3]. As expected, a voltage rise is produced by light illumination, and the Voc is dependence on the junction resistance.

**Literatures:**

1. Shang DS, Sun JR, Shi L, Shen BG (2008) Photoresponse of the Schottky junction Au/SrTiO3:Nb in different resistive states. Appl Phys Lett **93**:102106

2. Shang DS, Sun JR, Shi L, Wang ZH, Shen BG (2008) Resistance dependence of photovoltaic effect in Au/SrTiO3:Nb (0.5 wt%) Schottky junctions. Appl Phys Lett **93**:172119

3. Shang DS, Sun JR, Shen BG, Wuttig M (2013) Resistance switching in oxides with inhomogeneous conductivity. Chin. Phys. B **22**: 067202
